# The genetic architecture of growth and fillet traits in farmed Atlantic salmon (*Salmo salar*)

**DOI:** 10.1186/s12863-015-0215-y

**Published:** 2015-05-19

**Authors:** Hsin Yuan Tsai, Alastair Hamilton, Derrick R Guy, Alan E Tinch, Stephen C Bishop, Ross D Houston

**Affiliations:** The Roslin Institute and Royal (Dick) School of Veterinary Studies, The University of Edinburgh, Midlothian, EH25 9RG UK; Landcatch Natural Selection Ltd., 15 Beta Centre, Stirling University Innovation Park, Stirling, FK9 4NF UK

**Keywords:** Atlantic salmon, Aquaculture, Marker-assisted selection, Quantitative trait loci mapping, QTL mapping, *Salmo salar*

## Abstract

**Background:**

Performance and quality traits such as harvest weight, fillet weight and flesh color are of economic importance to the Atlantic salmon aquaculture industry. The genetic factors underlying these traits are of scientific and commercial interest. However, such traits are typically polygenic in nature, with the number and size of QTL likely to vary between studies and populations. The aim of this study was to investigate the genetic basis of several growth and fillet traits measured at harvest in a large farmed salmon population by using SNP markers. Due to the marked heterochiasmy in salmonids, an efficient two-stage mapping approach was applied whereby QTL were detected using a sire-based linkage analysis, a sparse SNP marker map and exploiting low rates of recombination, while a subsequent dam-based analysis focused on the significant chromosomes with a denser map to confirm QTL and estimate their position.

**Results:**

The harvest traits all showed significant heritability, ranging from 0.05 for fillet yield up to 0.53 for the weight traits. In the sire-based analysis, 1695 offspring with trait records and their 20 sires were successfully genotyped for the SNPs on the sparse map. Chromosomes 13, 18, 19 and 20 were shown to harbor genome-wide significant QTL affecting several growth-related traits. The QTL on chr. 13, 18 and 20 were detected in the dam-based analysis using 512 offspring from 10 dams and explained approximately 6–7 % of the within-family variation in these traits.

**Conclusions:**

We have detected several QTL affecting economically important complex traits in a commercial salmon population. Overall, the results suggest that the traits are relatively polygenic and that QTL tend to be pleiotropic (affecting the weight of several components of the harvested fish). Comparison of QTL regions across studies suggests that harvest trait QTL tend to be relatively population-specific. Therefore, the application of marker or genomic selection for improvement in these traits is likely to be most effective when the discovery population is closely related to the selection candidates (e.g. within-family genomic selection).

**Electronic supplementary material:**

The online version of this article (doi:10.1186/s12863-015-0215-y) contains supplementary material, which is available to authorized users.

## Background

Traditional selective breeding has rapidly improved economically important traits in aquaculture species, such as growth and disease resistance in aquaculture species [1]. Atlantic salmon have been more extensively studied than most other aquaculture species due to its high economic value and the significant scientific interest in salmonid species [2]. However, the genetic factors affecting some complex traits of economic importance, such as size, morphology and composition, are not yet well known. The limitations to detecting and defining these genetic factors may include a previous lack of genomic resources, the polygenic nature of the traits in question, and the relatively recent whole genome duplication (e.g. [3, 4]) in the salmonid lineage.

**Fig. 1:**
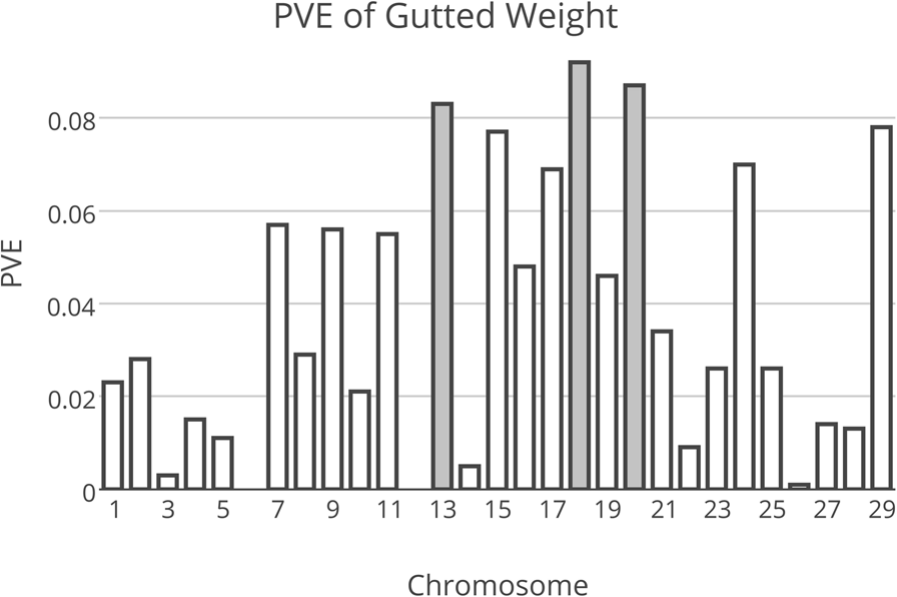
The distribution of PVE according to chromosome in the sire-based analysis for the representative weight trait of gutted weight. Gray represents the chromosome showing genome-wide significance (p < 0.05) in sire-based analysis. Chromosome 20 also showed chr-wide significance in dam-based analysis (p < 0.05)

Genomic resources for salmonids are rich in comparison to most aquacultural species [5]. Benefitting from the development of next generation sequencing (e.g. [6]), abundant genetic markers have been discovered in most salmonid species (e.g. [7–10]). Many other genomic resources and salmonid-specific databases are available, e.g. the Genomics Research on All Salmon (GRASP, http://web.uvic.ca/grasp/) and SalmonDB (http://salmondb.cmm.uchile.cl/). Furthermore, the genomes of rainbow trout [3] and Atlantic salmon [2] have been sequenced and assembled, which provide reference sequences for genomic studies of these and other salmonid species [11].

Understanding the genetic basis of phenotypic variation is a fundamental goal of biological research. Quantitative genetic analysis has been widely used to apportion variation in the traits of interest into genetic and environmental factors [12]. A further goal is to ascertain the genetic architecture of these traits, and quantitative trait loci (QTL) mapping is useful for this purpose. This approach has been widely applied in most farmed animal and plant species to improve genetic breeding programs [13–16]. To date, QTL mapping relating to the growth performance of farmed salmonid species have been undertaken in Atlantic salmon [17–21], Coho salmon [22, 23], Arctic char [24], Chinook salmon [25] and Rainbow trout [26, 27]. The loci associated with these apparently polygenic growth traits tend to vary between studies, which may reflect population differences or gene by environment interaction.

Traits of economic interest in aquaculture species include those pertaining to the efficient production of high quality fillets. As such, overall growth rate is important, alongside the relative proportion of particular components of the fish (fillet, guts, and head, etc.). Ultimately, fillet weight is a key economic trait, and variation in this characteristic significantly depends on the proliferation and composition of white and red muscle. Muscle cell development and proliferation are part of a complex regulatory process and intricately linked with the development of the skeleton. These processes are typically controlled by networks involving many genes and biological pathways [28]. As such, a polygenic architecture of variation in this trait may be expected. Previous studies have shown that the less desirable parts of Atlantic salmon (e.g. head weight and vertebral weight) have a significant positive correlation with desirable traits such as harvest and fillet yields [29]. By detecting and selecting haplotypes at specific QTL, it may be possible to improve the proportion of fillet within the fish for any given growth rate (albeit caution should also be applied to ensure overall wellbeing and robustness of the fish).

The objective of this study was to detect and characterize QTL affecting growth and fillet characteristics in farmed salmon, using SNP markers genotyped in several large families reared under commercial aquaculture conditions. Due to the lower recombination rate observed throughout much of the genome in male salmon, compared to female salmon [30], the efficiency of QTL detection is increased by using a two stage analysis. In this strategy, QTL are first detected in a sire analysis using few markers per chromosome, and the chromosomes harbouring significant QTL are then genotyped for additional markers and analysed using dam mapping parents. Here, we use this approach with the overall target of improving understanding of the genetic regulation of growth and fillet characteristics in Atlantic salmon, and providing candidate regions for potential application in marker-based selection to capture within-family variation in these traits.

## Methods

### Animals and phenotype measurement

A commercial salmon population comprising 198 full-sibling families derived from 136 sires and 198 dams (Landcatch Natural Selection, Ormsary, UK) was utilized in this experiment. Details of this population have been previously published [31–33]. Briefly, approximately 5000 fish were harvested at ~3 years of age and measured for overall and component weight traits: harvest weight (kg), gutted weight (kg), deheaded weight (kg), fillet weight (kg), gutted yield (%), fillet yield (%), head weight (kg), gut weight (kg), body waste weight (kg) and total waste weight (kg), fat percentage [% as estimated using a Torry Fatmeter (Distell Ltd, Aberdeen, Scotland)]; and fillet color [assessed visually using the Roche SalmoFan scale (Hoffmann-La Roche, U.K.), ranging from 20 (Yellow) to 34 (Red)]. Details of trait measurements at harvest are given in Powell *et al.* [29]. A fin clip sample of each fish was retained for DNA extraction. All animals were reared and harvested in accordance with all relevant national and EU legislation concerning health and welfare. Landcatch are accredited participants in the RSPCA Freedom Foods standard, the Scottish Salmon Producers Organization Code of Good Practice, and the EU Code-EFABAR Code of Good Practice for Farm Animal Breeding and Reproduction Organizations. The traits of fat percentage and gut weight were log_10_ transformed to approximate a normal distribution. Two generation pedigree records were available for all fish and the sex of the offspring was not observable at harvest and processing. Heritability estimates for some of the traits have been estimated previously in the larger population from which the QTL families were sampled [31, 32]. For gut, head, waste and total waste weight, the polygenic heritability was estimated in this larger population using a simple animal model, *Y*_*ij*_ *= μ + A*_*i*_ *+ e*_*ij*_, where *Y*_*ij*_ is the trait value measured in the individual *i*, μ is the overall mean value of the trait, *A*_*i*_ is the additive genetic effect of the individual based on the pedigree information and *e*_*ij*_ is the residual error. The heritability for each of the traits was estimated using the above model, and the procedure was described in Tsai *et al.* [32].

### SNP marker selection and genotyping

To account for the large differences in recombination rate between male and female salmon, a two-stage QTL detection and mapping strategy was employed [30, 34]. Stage 1 used sire mapping parents (low recombination), with few markers per chromosome to detect chromosomes containing putative QTL. Stage 2 used dam mapping parents, with a denser marker coverage, to confirm QTL on significant chromosomes and estimate QTL position. For stage 1, the 20 sires in the population with the most progeny were chosen for analysis (total n = 1695). The sparse panel of SNP markers described in Gonen *et al.* [35], largely taken from Moen *et al.* [36], were provided to LGC Genomics (Herts, U.K.) for the design of Kompetitive Allele Specific PCR (KASP) assays (see details at http://www.lgcgroup.com/products/kasp-genotyping-chemistry/kasp-technical-resources/#.VVUKo_1waM8) for genotyping. From these, a total of 51 informative SNPs, with one to three SNPs per chromosome, were genotyped in all 1695 offspring (Additional file 1: Table S1). Stage 2 aimed to confirm the QTL detected in stage 1 and to estimate their position on the chromosome. Therefore, stage 2 focused on three putative QTL-containing chromosomes (chr. 13, 18, and 20) detected in stage 1. Thirty additional segregating SNP markers (Additional file 1: Table S1) [9] were chosen to be positioned at regular (~10 cM) intervals across the candidate chromosome according to published linkage maps. As such, it was anticipated that this marker density would be sufficient to estimate approximate position of QTL on chromosomes. These SNPs were selected for genotyping in the ten dams with the largest number of offspring. A total of ten, eight and eight informative SNPs from chr. 13, 18 and 20 respectively were genotyped in the 512 offspring of these dams (which were a subset of the offspring genotyped offspring in stage 1).

### Linkage and QTL mapping

Sex-specific genetic maps were constructed using Crimap version 2.4 [37]. The ‘prepare’ option was used to create the input files (markers had previously been assigned to linkage groups based on a LOD score of >4.0), followed by the ‘build’ option to estimate marker order, and ‘fixed’ option to estimate the map distance between the markers. Where relevant, the ‘flipsn’ option was used to test different order permutations and determine the most likely marker order.

For both sire and dam based QTL detection, a two stage linear regression-based linkage analysis was performed using the GridQTL software [38]. The conditional probability of inheriting a particular haplotype from the sire or dam was inferred from the marker genotypes in all offspring, at 1 cM intervals. Subsequently, the trait value was regressed on the probability that a particular haplotype allele was inherited from the sire (stage 1) or the dam (stage 2). At each genomic location, the model containing a single QTL is compared to a model with no QTL resulting in an F Ratio statistic. The chromosome-wide significance thresholds for each trait were computed by permutation using 10,000 iterations per chromosome. With 29 chromosomes, the expected number of false positive was 1.45 at the 5 % significance threshold, and 0.29 at the 1 % significance threshold per genome scan respectively. The genome-wide thresholds were determined by applying the Bonferroni correction [39] to 29 independent chromosomes. In addition, in the stage 2 dam-based analysis, the confidence intervals for the QTL were estimated using bootstrapping with 10,000 permutations. In order to estimate the size of the effect of the significant QTL on the traits, the within-family variation explained by the QTL (PVE) was calculated using the following equation: h^2^_QTL_ = 4[1-(MSE_full_ / MSE_reduced_)] for sire-based analysis. For the dam-based analysis, because the dams were nested within sires (full-sibling families), the estimated equation was revised to h^2^_QTL_ = 2[1-(MSE_full_ / MSE_reduced_)], where the MSE_full_ is the mean square error of the performed model including the QTL and MSE_reduced_ is the model including the family mean only.

For traits related to the component weights of the fish, the QTL analyses were repeated including harvest weight as a covariate in the analysis. This was done to assess and distinguish QTL associated with an overall growth effect on the fish, versus QTL associated with proportional growth of specific components (e.g. fillet and waste, etc.)

## Results

Trait records of 1695 offspring derived from 20 sire families were obtained from a larger dataset of ~5000 salmon measured at harvest (~3 years old). The heritability of the weight traits was significant and consistent with previous estimated (h^2^ = 0.52 to 0.53). For the traits not previously analysed in this population (i.e. gut, head, waste and total waste weight) the heritabilities ranged from 0.15 to 0.32. Summary statistics from the QTL-mapping offspring and population-wide estimates of heritability for these traits are given in Table 1. The weight traits showed a high phenotypic and genetic correlation (Table 2) and fitting overall harvest weight as a covariate in the animal model reduced the estimated h^2^ for the component traits to 0.02 – 0.05 (although these were still significantly different from zero).

**Table 1:** Summary statistics and heritabilies for the phenotypes used in this study

Trait	Sample Size^†^	Mean (SD)	Heritability (SE) [29]
Harvest Weight	1524	2.57 (0.63)	0.52 (0.05)
Gutted Weight	1616	2.35 (0.58)	0.53 (0.05)
Gutted Yield	1447	0.92 (0.02)	0.04 (0.01)
Deheaded Weight	1604	2.06 (0.52)	0.52 (0.05)
Fillet Weight	1516	1.70 (0.42)	0.53 (0.05)
Fillet Yield	1363	0.66 (0.04)	0.05 (0.02)
Fat Percentage	1679	12.2 (5.58)	0.18 (0.03)
Fillet Colour	1322	29.0 (0.73)	0.14 (0.03)
Head Weight	1475	0.32 (0.08)	0.21 (0.03)^a^
Gut Weight	1447	0.42 (0.08)	0.30 (0.04)^a^
Body Waste Weight	1426	0.33 (0.12)	0.15 (0.02)^a^
Total Waste Weight	1422	0.65 (0.17)	0.32 (0.04)^a^

Gut weight (kg) = harvest weight - gutted weight; Head weight (kg) = gutted weight - deheaded weight

Waste weight (kg) = deheaded weight - fillet weight (weight of vertebrae and caudal fin); Total waste weight (kg) = head weight + body waste weight

^a^The heritability was estimated in this study and the used population was the same as Tsai *et al.* [32]

^†^: Only the number of individuals used in the calculation is shown, after removal of missing data

**Table 2:** Genetic and phenotypic correlation of traits using in this study

Genetic/Phenotypic	Harvest weight	Gutted weight	Gutted yield	Deheaded weight	Fillet weight	Fillet yield	Fat percentage	Fillet colour	Gut weight	Head weight	Body waste weight	Total waste weight
Harvest Weight	-	1.00	0.16	1.00	1.00	0.35	0.84	−0.17	−0.96	0.97	1.00	0.98
Gutted Weight	1.00	-	0.19	1.00	1.00	0.33	0.83	−0.20	−0.95	0.98	0.99	0.99
Gutted Yield	−0.02	0.06	-	0.19	0.20	0.53	0.05	−0.27	0.13	0.08	0.06	0.09
Deheaded Weight	0.98	0.98	0.06	-	0.99	0.37	0.83	−0.19	−0.95	0.97	1.00	0.98
Fillet Weight	0.97	0.97	0.05	0.97	-	0.41	0.82	−0.20	−0.95	0.95	1.00	0.98
Fillet Yield	0.02	0.06	0.31	0.08	0.27	-	0.21	−0.15	−0.21	0.09	0.23	0.19
Fat Percentage	0.41	0.41	0.04	0.41	0.42	0.07	-	−0.19	−0.82	0.76	0.84	0.80
Fillet Colour	−0.08	−0.08	−0.02	−0.07	−0.08	0.03	−0.06	-	0.10	−0.24	−0.13	−0.12
Gut Weight	−0.77	−0.72	0.56	−0.71	−0.72	0.12	−0.30	0.05	-	−0.94	−0.99	−0.96
Head Weight	0.61	0.62	0.04	0.47	0.92	−0.09	0.21	−0.11	−0.45	-	0.99	1.00
Body Waste Weight	0.62	0.61	0.09	0.63	0.41	−0.65	0.25	−0.04	−0.42	0.63	-	1.00
Total Waste Weight	0.83	0.83	0.08	0.83	0.67	−0.48	0.31	−0.07	−0.59	0.88	0.93	-

In total, 51 SNP markers dispersed over all 29 chromosomes were successfully genotyped in the parents and offspring. In the sire-based QTL mapping analysis, a total of 13 chromosomes showed suggestive evidence for a QTL (chromosome-wide p < 0.05), while four chromosomes showed a significant effect on growth-related traits at the genome-wide leve1 (chr. 13, 18, 19, and 20; Table 3, Figure 1). The QTL typically affected several of the weight measurements and, given the high phenotypic correlations between these traits (r ~ 0.97-1.00), it is plausible that these results reflect single pleiotropic QTL on each chromosome, rather than distinct linked QTL. Interestingly, when harvest weight was fitted as a covariate (as a proxy for an overall measure of growth), the QTL affecting the component traits on chr. 18, 19 and 20 were no longer significant, suggesting these QTL affect overall growth of the fish. In contrast, on chr. 13, most of the QTL effects for the component traits remained after fitting the covariate, suggesting putative proportional differences in the growth of components of the fish. In addition, four new QTLs (chr. 12, 22, 23, and 25) reached chromosome-wide significance in the sire-based analysis with the inclusion of harvest weight as a covariate in the analysis (Table 3). The proportion of within-family phenotypic variance explained (PVE) varied between 8 and 10 % for the genome-wide significant QTL in the sire-based analysis, suggesting QTL of moderate but not large effect in this population.

**Table 3:** Results of sire-based QTL mapping analysis and proportion of phenotypic variance explained by each chromosome

	Harvest Weight		Fillet Weight				Gutted Weight				Deheaded Weight				Fillet Yield			
					covariate^$^				covariate^$^				covariate^$^				covariate^$^	
Chr	F ratio	PVE	F ratio	PVE	F ratio	PVE	F ratio	PVE	F ratio	PVE	F ratio	PVE	F ratio	PVE	F ratio	PVE	F ratio	PVE
1	-	-	1.88*	0.047	-	-	-	-	-	-	-	-	-	-	-	-	-	-
6	-	-	-	-	-	-	1.96*	0	-	-	-	-	-	-	1.90*	n.a.†	1.89*	n.a.†
7	-	-	-	-	-	-	2.03*	0.057	-	-	2.07*	0.06	1.79*	0.037	-	-	-	-
9	-	-	2.04*	0.055	-	-	2.12*	0.056	-	-	2.26*	0.062	-	-	-	-	-	-
10	-	-	2.08*	0.053	-	-	-	-	-	-	-	-	-	-	-	-	-	-
11	2.10*	0.065	2.27*	0.077	2.26**	0.071	1.96*	0.055	-	-	1.91*	0.052	-	-	2.32*	n.a.†	2.31**	n.a.†
12	-	-	-	-	-	-	-	-	-	-	-	-	2.05*	0.078	-	-	-	-
13	2.49**	0.074	2.53**	0.075	1.90*	0.07	2.78**	0.083	-	-	2.67**	0.08	1.81*	0.034	2.37*	n.a.†	2.37**	n.a.†
15	2.12*	0.076	-	-	-	-	2.24*	0.077	-	-	2.43*	0.091	-	-	-	-	-	-
16	2.23*	0.06	-	-	-	-	2.06*	0.048	-	-	2.19*	0.055	-	-	-	-	-	-
17	2.22*	0.082	2.47*	0.096	-	-	2.10*	0.069	-	-	2.37*	0.085	-	-	-	-	-	-
18	2.59**	0.083	2.76**	0.092	-	-	2.89**	0.092	-	-	2.82**	0.089	-	-	-	-	-	-
19	2.00*	0.05	2.62**	0.078	-	-	2.00*	0.046	-	-	2.00*	0.049	-	-	-	-	-	-
20	2.66**	0.09	2.42*	0.077	-	-	2.76**	0.087	-	-	2.76**	0.09	-	-	-	-	-	-
21	-	-	-	-	-	-	1.72*	0.034	1.89*	0.114	-	-	-	-	-	-	-	-
22	-	-	-	-	-	-	-	-	-	-	-	-	1.95*	0.063	-	-	-	-
23	-	-	-	-	1.86*	0.033	-	-	-	-	-	-	-	-	-	-	-	-
24	2.52*	0.068	2.58*	0.07	-	-	2.66*	0.07	2.45*	n.a.†	2.84*	0.079	2.56*	0.066	-	-	-	-
27	1.77*	0.039	-	-	-	-	-	-	-	-	-	-	-	-	-	-	-	-
28	1.88*	0.043	-	-	-	-	-	-	-	-	-	-	-	-	-	-	-	-
29	-	-	1.97*	0.06	-	-	2.31*	0.078	-	-	2.32*	0.079	-	-	-	-	-	-
	Head Weight				Gut Weight					Body Waste Weight		SalmoFan Scale		Total Waste Weight				
			covariate^$^				covariate^$^									covariate^$^		
Chr	F ratio	PVE	F ratio	PVE	F ratio	PVE	F ratio	PVE		F ratio	PVE	F ratio	PVE	F ratio	PVE	F ratio	PVE	
1	1.76*	0.07	-	-	-	-	-	-		-	-	-	-	1.80*	0.068	-	-	
3	-	-	-	-	-	-	-	-		-	-	2.30*	0.088	-	-	3.20**	0.154	
6	2.12*	0.074	-	-	-	-	-	-		-	-	-	-	2.05*	0.078	-	-	
7	-	-	-	-	1.73*	0.044	1.72*	0.048		2.01*	0.063	-	-	2.49*	0.075	-	-	
9	2.05*	0.07	-	-	-	-	-	-		-	-	-	-	-	-	-	-	
11	1.96*	n.a.†	-	-	-	-	-	-		-	-	-	-	-	-	2.00*	0.099	
13	2.72**	0.072	-	-	-	-	-	-		-	-	-	-	2.44*	0.046	1.91*	0.052	
16	-	-	-	-	1.98*	0.044	-	-		-	-	-	-	-	-	-	-	
17	-	-	-	-	2.16*	0.093	-	-		-	-	2.11*	0.085	-	-	-	-	
18	2.11*	0.069	-	-	-	-	-	-		-	-	-	-	1.88*	0.047	-	-	
19	2.49**	0.068	-	-	-	-	-	-		-	-	-	-	2.22*	0.033	-	-	
20	2.02*	0.067	-	-	2.19**	0.063	-	-		-	-	-	-	1.99*	0.048	-	-	
21	-	-	1.72*	n.a.†	-	-	-	-		1.85*	0.03	-	-	1.76*	0.046	-	-	
22	-	-	1.90*	0.4	-	-	-	-		-	-	-	-	-	-	-	-	
24	2.24*	0.068	-	-	3.03**	0.094	3.13**	0.126		-	-	-	-	-	-	-	-	
25	-	-	1.97*	0.4	-	-	-	-		-	-	2.04*	0.067	-	-	-	-	

*: chromosome-wide significance at p < 0.05; **: genome-wide significance at p < 0.05; PVE: proportion of phenotypic variance for half-sib analysis

^†^: Due to the MSE_full_ value being equal to MSE_reduced_

$: Harvest weight was fitted as covariate

Three of the genome-wide significant QTL in the sire-based analysis (chr. 13, 18, and 20) were tested in a dam-based analysis using 512 offspring from ten dams, and a denser SNP marker map of the significant chromosomes (Additional file 1: Table S1). The genome-wide significant QTL affecting gutted, deheaded and total waste weight on chr. 20 was confirmed in the dam-based analysis and mapped to a best estimated position of 21, 19 and 14 cM respectively, although the 95 % confidence intervals encompassed the entire linkage map for this chromosome (Table 4). The evidence for QTL on chr. 13 and 18 was not as strong in the dam-based analysis, with only gut weight (chr. 13) and gutted weight (chr. 18) showing chromosome-wide significance (in the analysis with harvest weight included as a covariate). For the chr. 20 QTL, there were three sires and three dams segregating for a QTL affecting at least one weight trait, and the average size of the allelic substitution effect for deheaded weight of the salmon in segregating parents was consistent across all segregating parents, with an average effect of 620 grams (Table 5).

**Table 4:** Results of dam-based QTL mapping analysis and proportion of phenotypic variance explained for significant trait/chromosome combinations

Chr	Trait	Dam F-ratio	PVE	Average QTL position (cM)	95 % C.I. for QTL Position (cM)
20	Gutted Weight	2.48*	0.06	20.8	0.0 - 43.0
	Deheaded Weight	2.71*	0.07	19.4	
	Total Waste Weight	2.35*	0.06	14.0	
	Body Waste Weight	2.18*	0.06	12.4	0.0 - 40.0
13^†^	Gut Weight	2.49*	0.07	42.0	0.0 - 64.0
18^†^	Gutted Weight	2.62*	0.07	20.2	0.0 - 39.0

*: chromosome-wide significance at p < 0.05; ^†^: QTLs found in the analysis fitting harvest weight as covariate

PVE: proportion of phenotypic variance for full-sib analysis

**Table 5:** The QTL effect on growth traits and associated absolute T values in segregating individual parents for the significant QTL at chr. 20

Sire-based analysis	Traits	QTL effect estimate (SE)^a^ (g)	Absolute T value
J9L2M0088	Harvest Weight	−580 (170)	3.43
	Fillet Weight	−430 (110)	4.01
	Gutted Weight	−650 (150)	4.41
	Deheaded Weight	−580 (130)	4.41
	Head Weight	−80 (20)	3.61
	Total Waste Weight	−140 (50)	2.98
J9L2M0091	Harvest Weight	−360 (160)	2.19
J9L3M3080	Total Waste Weight	170 (70)	2.29
**Dam-based analysis**			
J9L2F0144	Gutted Weight	570 (170)	3.36
	Deheaded Weight	480 (150)	3.28
	Total Waste Weight	140 (50)	2.53
J9L2F1295	Gutted Weight	590 (250)	2.35
	Deheaded Weight	480 (200)	2.35
	Body Waste Weight	200 (60)	3.56
	Total Waste Weight	270 (80)	3.52
J9L2F0695	Deheaded Weight	−940 (400)	2.32

^**a**^The sign + or – is arbitrary when compared across families but indicates the direction of the allelic effect within families (e.g. an allele decreasing harvest weight in sire J9L2M0088 also decreased fillet, gutted, deheaded, head and total waste weight)

## Discussion

In this study, the genetic basis and architecture of growth-related traits was investigated in a large commercial population of Atlantic salmon using a two-stage QTL mapping approach. All traits measured showed significant evidence for heritability and significant weight-related QTLs were observed on chr. 13, 18, 19 and 20 in the sire-based analysis. These QTL typically affected several of the weight measurements taken at harvest, which reflects the high positive correlation between these traits and suggests that their effect is related to overall size of the fish. However, the QTL on chr. 13 may have effects on the weight of components of the fish independent of an overall growth effect, as indicated by an analysis including harvest weight as a covariate.

A QTL affecting several of the growth-related traits on chr. 20 was confirmed in the dam-based analysis. This chromosome has previously been shown to harbor QTL affecting body weight of Atlantic salmon at younger age (10 months; [16]). However, a comparison of the QTL detected in the current study with those observed in previous studies (Table 6) shows that, even amongst populations of salmon measured at similar age, QTL tend to be rather population-specific. This may reflect differing underlying quantitative trait nucleotide affecting growth of the populations, genotype by environment interaction, or simply that a proportion of QTL identified in most studies are likely to be ‘false positives’. The weight traits measured at harvest had high positive genetic and phenotypic correlations (r ~ 0.97-1.00 in phenotypic and ~0.99-1.00 in genetic correlation), and this is generally reflected in the QTL results, because individual QTL tended to affect the weight of several components of the fish. This is a phenomenon observed in several other studies (e.g. [18]) and suggests that improvement of the growth of all components of the fish in breeding programs can be made by simply measuring overall harvest weight. This will improve harvest weight, the most important trait, but is likely to also improve potentially undesirable traits such as gut weight. Achieving different rates of gain in individual components of the fish using QTL or conventional family-based selection is likely to be challenging and may require more detailed or accurate measures of these component traits. However, the existence of QTL affecting fillet weight seemingly independent of overall harvest weight (e.g. chr. 11) suggests that there are potentially some genes affecting component traits partially independently of harvest weight that could be targets for further study.

**Table 6:** Comparison of harvest weight QTL chromosomes in Atlantic salmon from this and previous studies

	Gutierrez *et al.* [17]		Baranski *et al.* [18]	Houston *et al.* [19]	This study	
					Sire	Dam
Chr/Age	~27 months	~38 months	~36 months	~30 months	~36 months	
1		C	C			
2	G	C		C/G		
3				C		
4			G			
5	C	C	G			
6				C		
7			C			
8	C			C		
9	C					
10	C		G			
11			C		C	
12						
13			C	C/G	G	
14						
15	C	C			C	
16		C	C		C	
17					C	
18			C		G	C
19	C				C	
20					G	C
21	C			C		
22			C			
23			C			
24					C	
25		C	C			
26		G				
27		C			C	
28					C	
29	C		C			

C: chromosome-wide significance; G: genome-wide significance

Atlantic salmon are closely related to rainbow trout and previous studies in trout have reported several QTLs affecting body mass [25, 40-41]. There was some overlap between these QTL and the genome-wide significant QTL identified in the current study, in particular for body mass QTL mapped to trout chromosomes 1q and 16q/12p [26], chr. 9p and 21p [42] and chr. 16q [40], which correspond to chr. 13 and 18 in salmon. In addition, corresponding QTL regions showing chromosome-wide significance with body weight were also discovered between Chinook salmon (chr. 25) [25] and Atlantic salmon (chr. 28) (this study). These results raise the possibility that some of the QTL affecting complex growth traits may be conserved across salmonid species. However, clearly some overlap between studies will occur by chance and the likelihood of the underlying QTL being common in both species will become more apparent with further studies and a finer mapping resolution. The confidence intervals associated with the QTL in the current study were large which precluded the meaningful identification of potential underlying candidate genes. However, known candidate genes explaining a small percentage of variation in growth in this population (*myostatin* [31] and *IGF1* [32]) do not coincide with the QTL identified here.

The size of the QTL effects in the current study was typically around 5-9 % and 6-7 % of the within-family phenotypic variance in the sire and dam-based analysis respectively. While this may be an overestimate due to the Beavis effect [41], it is certainly plausible that markers linked to these QTL may be of use in selective breeding programs. However, the confidence intervals were large and this indicates that while the two-stage mapping approach employed appears to be effective at detecting QTL, the fine mapping to smaller chromosome regions in the dam analysis may benefit from additional markers. The results of this and other studies support the hypothesis that complex traits such as weight are polygenic, which may reflect the involvement of diverse regulation pathways related to energy balance, muscle cell proliferation and skeletal growth. The fact that the proportion of variation explained by the QTL is smaller than in previous studies (e.g. [19]) is probably due to the large sample size of the current study (i.e. ~1700 offspring for the sire-based analysis), and hence potentially more reliable estimates of QTL effect size [41]. Further, the two-step approach provided some degree of within-study validation for the detected QTL on chr. 18. The traits of most commercial interest in salmon production, such as fillet weight were affected by the QTL on chr. 13, 18, 19, and 20 (genome-wide significance) in the sire-based analysis. Notably, except chr. 19 in sire-based analysis - for which further study may be merited - all of these QTL regions showed a significant effect on gutted weight and deheaded weight.

No QTL affecting fat content were detected in our study. Interestingly, components of fat content of salmon, such as n-3 long chain polyunsaturated acid, are highly heritable [43]. Therefore, perhaps more consideration could be given to the investigation of the genetic architecture of the specific components of the fat content of the fillet, as opposed to a more crude overall measure of fat levels. Naturally, this refinement of phenotype would incur a greater cost. In addition, only three QTL (chr. 3, 7 and 26) were shown to affect fillet colour at the chromosome-wide significance level. Chromosomes 3 and 26 have previously been suggested to harbor QTL associated with fillet colour traits [18]. The heritability of this trait is relatively low in this study (h^2^ ~ 0.1 - 0.2) when compared with weight related traits in Atlantic salmon [44], although recently published studies have given higher heritabilities [45] and fillet color has been suggested to show a significant association with a single locus SCAR marker [46]. It has also been suggested that fillet colour is positively correlated with overall body weight in farmed Coho salmon (r ~ 0.4 ± 0.5) [46] and Atlantic salmon (r ~ 0.49) [47]. This may be related to the inclusion of dietary additives such as astaxanthin, canthaxanthin and carotenoid, which are included in feed to enhanced fillet pigmentation [48]. As such, protein/muscle gains may be accompanied by an associated increase in colour additives. However, we did not observe a correlation between harvest weight and fillet colour in our study. In part, this may be due to a lack of fillet colour variation observed in the population (coefficient of variation ~ 0.025). Of the putative colour QTL in the current study, only chr. 17 showed some evidence for an effect on growth-related traits, while chr. 3 and 26 were associated with fillet colour independent of the other traits measured. Given the economic importance of this trait, further study of these putative QTL and other aspects of the genetic regulation of colour are merited.

Marker-assisted selection has been applied in the salmon aquaculture industry for several traits, the foremost example being resistance to the Infectious Pancreatic Necrosis virus [34, 49, 50]. However, the genetic architecture of resistance to this disease was unusually monogenic, with a single QTL explaining most of the genetic variation. For more typical complex traits such as growth or fillet component traits, the optimal use of markers in selective breeding programs has yet to be established. Clearly, the advantages of using markers in selection for aquaculture are maximal where the traits are difficult or impossible to measure on the selection candidates themselves, and some of the harvest traits fall into this category. However, due to the lack of large-effect QTL and the putative population-specificity of those QTL, it is unlikely that QTL-targeted, across population marker-assisted selection will be a highly effective tool for breeding. With the recent development of high density SNP arrays (e.g. [7]), genomic selection may be a more effective (albeit expensive) means of capturing variation at QTL of small effect, but is likely to be the most effective when the training and selection population are closely related. Within family genomic selection using lower marker density may be a more cost-effective method of capturing the within-family genetic variation associated with QTLs that are relatively population-specific [51]. The large full-sibling family sizes and routine sib-testing in salmon breeding schemes makes such approaches feasible and powerful.

## Conclusions

This study investigated the genetic basis of traits measured at harvest in a large commercial population of Atlantic salmon. The traits showed significant heritability and four genome-wide significant QTL were identified on chr. 13, 18, 19 and 20. The QTL on chr. 20 had relatively large effects on several weight-related traits that were consistent in the sire and dam analysis. The abundant putative QTLs provide a broad view of the genetic architecture of body weight and component traits in salmon. It is likely that weight traits in salmon are controlled by a finite number of partially population-specific loci of moderate-effect, in addition to a large polygenic component. These factors should be accounted for when considering the optimal methods of applying genomic markers in selective breeding programs.

## Additional file

Additional file 1: Table S1. Details of the SNP markers used in this study.

## Abbreviations

QTL: Quantitative trait loci
KASP: Kompetitive Allele Specific PCR
PVE: Proportion of phenotypic variance
SNP: Single nucleotide polymorphism
LOD: Logarithm of the odds
*IGF1*: Insulin-like growth factor 1
