# Supplementary material for: The genetic architecture of growth and fillet traits in farmed Atlantic salmon (Salmo salar)
Source: BMC Genet. 2015 May 19;16:51. doi: 10.1186/s12863-015-0215-y (PMC4436873; doi:10.1186/s12863-015-0215-y)
Supplement: Additional file 1: Table S1. — Details of the SNP markers used in this study. [file 12863_2015_215_MOESM1_ESM.docx]

Supplementary Table 1. Details of the SNP markers used in this study.

| **SNP Marker ID used in Stage 1** [36] | **SNP Marker ID used in Stage 2** [9] |
| --- | --- |
| Ssa0114ECIG | **Chr. 13** |
| Ssa0145ECIG | ESTNV_36249_105 |
| Ssa0051ECIG | ESTNV_31779_190 |
| Ssa0014ECIG | GCR_hBin9111_Ctg1_244 |
| Ssa0235ECIG | ESTNV_35182_980 |
| Ssa0211ECIG | GCR_cBin42784_Ctg1_133 |
| Ssa0148ECIG | GCR_cBin8175_Ctg1_227 |
| Ssa0204ECIG | ESTNV_35620_594 |
| Ssa0012ECIG | GCR_cBin30085_Ctg1_133 |
| Ssa0130ECIG | GCR_cBin10604_Ctg1_137 |
| Ssa0081ECIG | GCR_cBin42908_Ctg1_216 |
| Ssa0203bECIG | **Chr. 18** |
| Ssa0093ECIG | ESTNV_32294_424 |
| Ssa0126aECIG | ESTNV_37407_341 |
| Ssa0137ECIG | GCR_cBin3648_Ctg1_155 |
| Ssa0181ECIG | ESTV_21085_409 |
| Ssa0252ECIG | GCR_cBin43669_Ctg1_203 |
| Ssa0190ECIG | GCR_cBin8069_Ctg1_204 |
| Ssa0142ECIG | GCR_cBin1260_Ctg2_204 |
| Ssa0076ECIG | ESTNV_24515_297 |
| Ssa0257ECIG | **Chr. 20** |
| Ssa0214ECIG | ESTNV_31164_1708 |
| Ssa0122aECIG | GCR_cBin10764_Ctg1_187 |
| Ssa0176ECIG | ESTNV_34684_693 |
| Ssa0213ECIG | GCR_cBin13953_Ctg1_112 |
| Ssa0207ECIG | GCR_hBin3043_Ctg1_167 |
| Ssa0064ECIG | ESTV_15139_351 |
| Ssa0096ECIG | ESTV_14071_338 |
| Ssa0080ECIG | ESTNV_31685_978 |
| Ssa0245ECIG |  |
| Ssa0136ECIG |  |
| Ssa0158ECIG |  |
| Ssa0109ECIG |  |
| Ssa0117bECIG |  |
| RAD010201 |  |
| Ssa0139ECIG |  |
| Ssa0085ECIG |  |
| Ssa0005ECIG |  |
| Ssa0196bECIG |  |
| Ssa0004ECIG |  |
| Ssa0171aECIG |  |
| Ssa0047bECIG |  |
| Ssa0168ECIG |  |
| Ssa0023ECIG |  |
| Ssa0192ECIG |  |
| Ssa0201ECIG |  |
| Ssa0172ECIG |  |
| Ssa0087ECIG |  |
| Ssa0067ECIG |  |
| Ssa0016ECIG |  |
| Ssa0111bECIG |  |
